# Emerging scaffold- and cellular-based strategies for brain tissue regeneration and imaging

**DOI:** 10.1007/s44164-022-00013-0

**Published:** 2022-03-17

**Authors:** Maurice N. Collins, Fernanda Zamboni, Aleksandra Serafin, Ane Escobar, Romain Stepanian, Mario Culebras, Rui L. Reis, Joaquim M. Oliveira

**Affiliations:** 1 School of Engineering and Bernal Institute, University of Limerick, Limerick, Ireland; 2 SFI AMBER, University of Limerick, Limerick, Ireland; 3 3B’s Research Group, I3Bs–Research Institute on Biomaterials, Biodegradables and Biomimetics, Headquarters of the European Institute of Excellence On Tissue Engineering and Regenerative Medicine, AvePark, Parque de Ciência E Tecnologia, Zona Industrial da Gandra, University of Minho, 4805-017 Barco, Guimarães Portugal; 4 ICVS/3B’s-PT Government Associate Laboratory, Braga, Guimarães Portugal

**Keywords:** Central nervous system, Brain, Biomaterials, Carriers, Cells, Imaging, Growth factors, Tissue regeneration

## Abstract

Stimulating brain tissue regeneration is a major challenge after central nervous system (CNS) injury, such as those observed from trauma or cerebrovascular accidents. Full regeneration is difficult even when a neurogenesis-associated repair response may occur. Currently, there are no effective treatments to stimulate brain tissue regeneration. However, biomaterial scaffolds are showing promising results, where hydrogels are the materials of choice to develop these supportive scaffolds for cell carriers. Their combination with growth factors, such as brain-derived neurotrophic factor (BDNF), basic fibroblast growth factor (bFGF), or vascular endothelial growth factor (VEGF), together with other cell therapy strategies allows the prevention of further neuronal death and can potentially lead to the direct stimulation of neurogenesis and vascularisation at the injured site. Imaging of the injured site is particularly critical to study the reestablishment of neural cell functionality after brain tissue injury. This review outlines the latest key advances associated with different strategies aiming to promote the neuroregeneration, imaging, and functional recovery of brain tissue.

**Graphical abstract:**

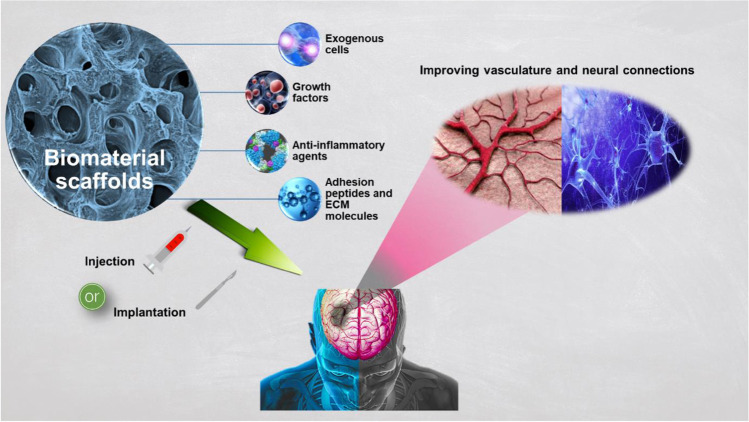

## Introduction

Neural injury is considered one of the most catastrophic, permanent, and untreatable types of damage to human tissue. Neural tissue death can be caused from trauma, cerebrovascular accidents, and degenerative diseases affecting both peripheral (PNS) and central nervous system (CNS) [1]. The result of such catastrophic injuries, originated from neurogenerative diseases, such as Parkinson, Alzheimer, and Huntington, or from brain injuries associated with stroke and spinal cord damage, is highly incapacitating [2]. After brain injury, cells die, often leading to functional and cognitive limitations [3], the quality of life of the patient is compromised, and this in turn increases the cost to healthcare systems [4].

The nervous system, consisting of the CNS and PNS, enables communication and coordination throughout the body. Cellular signals are received and processed, and cells are signalled for a response to external inputs [5, 6].

The PNS is a network of nerves that originate in the CNS and extend to all body parts and can be categorised into two main PNS systems—somatic and autonomous. The somatic system carries both afferent and efferent fibres while the autonomous automatically regulates organs and homeostasis [7]. The PNS acts as sensory and motor message carriers to and from the CNS into the external parts of the body such as glands and muscles [5].

Neurons, the building blocks of the nervous system, are classified as afferent, efferent, and interneurons based on function. Afferent neurons carry signals from receptors to the CNS. Efferent neurons, on the other hand, carry signals from the CNS throughout the body, while interneurons can carry out both functions [6, 8]. Neurons consist of dendrites, axons, and the supporting glial cells. Axons carry the electrical signals largely by means of surrounding myelin sheaths and Schwann cells, together composing nerve fibres and fascicles by means of nerve fibre bundles, though unmyelinated nerve fibres are also prevalent. The presence of the myelin sheath allows for faster signal speeds across the nerve [9].

The nervous system may be damaged at both the CNS and PNS level, arising from factors such as ischemic, chemical, or mechanical damage. Transection of nerves may also occur, interrupting cell–cell communication or disrupting the blood-nerve barriers [10]. Interestingly, the PNS has an inherent ability to regenerate axons when minor damage occurs. However, the CNS does not possess this capability [11]. However, recent advances in tissue engineering and regenerative medicine (TERM) have potential to offer the restoration of motor, sensory, and cognitive function [12].

Traumatic brain injury (TBI) can happen in several forms varying the degree of alteration, ranging from loss of consciousness to a comatose state and ultimately death [13]. When a TBI occurs, patients often lose memory of events that took place prior or after the injury, can suffer from neurologic deficits such as weakness, loss of balance, or altered vision, and may exhibit mental confusion, disorientation, or slowness of thinking, amongst other symptoms [14]. The two leading causes of TBI are vehicle accidents and falls [15], while a TBI can also be associated with the development of ischemic and haemorrhagic stroke, which results in neuronal tissue loss [16].

This tissue loss may subsequently lead to irreversible neurological dysfunction evidenced by the loss of functional abilities, which usually means patients are unable to live independently [17] or may even lead to death. For example, in the USA, the main incidence peak occurs at around 0–4 years in the adolescence and young age at 15–24 years, and in the elderly at 65 years and older [18]. In 2016, 5.5 million deaths were reported worldwide due to ischemic and haemorrhagic stroke [19]. About 80% of patients suffer from ischemic stroke due to blood vessels occlusion, whereas the remaining 20% are associated with haemorrhagic stroke, which consists of blood vessel rupture [20].

The pathogenesis of TBI is a complex and multi-step process. The initial cause consists of an external impact to the brain, and this leads to a cascade of events involving molecular, chemical, and inflammatory responses that are concurrent with brain damage [13]. Neuroinflammation can help to facilitate tissue repair, but is transient and self-limited, and it leads to a chronic inflammatory state in which the tissue degenerates [21]. Neurological damage results in glial scar tissue formation involving reactive astrocytes, macrophages, and dead neurons. Indeed, a traumatic event or ischemic process may lead to the disruption of the blood–brain barrier (BBB), which can provoke leakage of haematogenous cells into the neuronal tissue, cerebral oedema, and neuroinflammation [22]. The inflammatory response itself involves leukocytes, lymphocytes, and pro‐inflammation and anti‐inflammation macrophages, M1 and M2 macrophages, respectively, induced by cytokines [23]. Chemokines (CCL-2), interleukin (IL-6), and tumour necrosis factor (TNF-α) are also involved and they attract peripheral cells to the injured site of the brain, which release signalling factors that activate additional microglia, such as astrocytes, which can cause further trauma [24]. This can lead to cell and extracellular matrix (ECM) loss, creating a cavity and scar tissue in the glia. Glial scar tissue has detrimental effects on axonal regrowth, but can be beneficial by restraining cavity formation and plays a role in reconstituting the BBB [25]. There are two main areas in the adult brain that are known to produce new neurons: the subventricular zone (SVZ), in the lateral ventricles, and the subgranular zone (SGZ), in the hippocampus dentate gyrus [26]. This discovery has opened new possibilities to repair brain damages taking advantage of adult neurogenesis [27]. Astrocytes are the most important regulators in adult neurogenesis, and they are the primary source of proteins that regulate neural stem cells (NSCs) proliferations and differentiation as they secrete bone morphogenetic proteins and WNT proteins [28].

The pathophysiological mechanism after suffering a TBI leads to very heterogeneous injuries. Magnetic resonance imaging (MRI) is utilised for in vivo diagnosis of brain injury through risk assessment and biomarker evaluation [14]. Neuronal death is associated with loss of BBB integrity causing glutamate release, excessive production of reactive oxygen species, glucose decrease, and further loss of neurovascular functions [29]. Neuronal damage following TBI tends to lead to quick primary neuronal death [30]. However, neuroprotection strategies such as glutamate modulation [31], proteases calpains [32] and caspases inhibition [33], hypoxic preconditioning [34, 35], erythropoietin (EPO) use [36], or even astrocytes [37] show great promise in primary neuron rescue. Although there have been great advances in cell therapy for brain repair after injuries caused by a stroke using stem cells, such as foetal cells, induced pluripotent stem cells (iPSCs), embryonic stem cells (ESCs), or mesenchymal stem cells (MSCs), there are still hurdles in translating cell therapy research from trials to the clinic [38]. In a tissue engineering context, on the other hand, capturing the complex 3D network of the brain through in vitro models for TBI still requires refinements of the current existing models [39], such as the inclusion of microglial components to TBI models [40].

In this context, a detailed review of the literature highlighting recent achievements and challenges encountered in the development of tissue engineering strategies for brain tissue repair and MRI will be presented. Scaffolds play a key role as a support for cells to anchor and regenerate the injured site of the brain [41]. They need to match the environments from a biochemical and biophysical aspect and stimulate the infiltration of cells. Mechanics also play an important role with cell sensing influencing outcomes.

In this review, a compilation of the most recent discoveries and findings, associated with the latest approaches for neuroregeneration, will be discussed. Finally, this review will cover the topics of biomaterials selection, structural-function properties of scaffolds, immobilisation of growth factors, and the role of imaging that are particularly critical to the reestablishment of neural cell functionality after brain tissue injury.

## Current clinical treatments for brain tissue regeneration

Current treatments after a neural tissue loss are focused on reducing secondary effects and rehabilitative therapies rather than direct neurogenesis stimulation. Physical stimulation methods such as treadmill training [42] in combination with transcranial magnetic stimulation [43] or the use of robotic assistive devices [44] are the most common strategies amongst rehabilitative therapies. Pharmacologic interventions like amphetamine stimulation have also been reported, but they showed negative secondary effects for patients [45]. Regarding alternative therapies, virtual reality and acupuncture have shown beneficial in rehabilitation after brain tissue loss as a consequence of a stroke [46, 47]. Food and Drug Administration (FDA) approved thrombolytic therapies using intravenous alteplase (IV-tPA). It has shown good outcomes over nine trials for ischemic stroke but its main drawback is that it must be administered within 3 or 4.5 h following stroke, which is not possible for every patient [48, 49]. Endovascular thrombectomy has achieved similar outcomes to IV-tPA despite vascular access complications [50]. Table 1 summarises ongoing and already completed clinical trials over the last 10 years. They are discriminated in treatments for tissue loss, after a stroke has occurred, and TBI.

**Table 1:** Recent clinical trials focused on neuroregeneration. From ClinicalTrials.gov

Start/end or expected end date	Phase	Study ID	Country	Treatment	Final results or current outcomes
*Stroke-associated TBI*					
2013/2017	-	NCT01852201	USA	IV-tPA	Good functional outcomes
2009/2015	1	NCT00859014	USA	Bone marrow mesenchymal stem cells (BMSCs)	Serious adverse events and no real improvements
2006/2008	2	NCT00362414	USA	Human chorionic gonadotropin (beta-hCG) + EPO	Safety assessed
2012/2015	2	NCT01643902	USA	IV-tPA	No haemorrhage observed
2018/2020	1	NCT03570450	France	Adipose-derived SCs	Not available yet
2018/2022	2	NCT03629275	UK	NSCs	Not available yet
*Traumatic brain injury*					
2009/2016	3	NCT00987454	New Zealand	EPO	No improvements
2010/2016	2	NCT01212679	China	NGF	Not available yet
2012/2016	1 & 2	NCT01575470	USA	BMMNC	Not available yet
2006/2014	2 & 3	NCT00313716	USA	EPO	No improvements

## Approaches for neuroregeneration promotion and imaging

The ECM of brain is composed of proteoglycans that contain a lectin domain and a hyaluronic acid (HA)-binding domain, heparan sulphate (HS), and laminin [51]. More specifically, CS proteoglycans are attached to a hyaluronan backbone. All cell types found in the brain: neurons, astrocytes, oligodendrocytes, and microglia, produce these molecules, whereas fibronectin and collagen I are not native to brain tissue [52]. However, all these molecules are altered after TBI in terms of distribution and concentration [53]. Replicating the ECM with natural or synthetic polymer scaffolds is a key strategy to provide a specific environment for cell migration, survival, and differentiation. These scaffolds are temporal structures in the brain, they biodegrade and resorb, and can be either surgically implanted or injected.

### Scaffolds for brain tissue regeneration

For the successful development of an engineered brain tissue, the selection of an appropriate structure that will mimic, as closely as possible, the native ECM is of utmost importance so that it will generate substantial neuron outgrowth and vascular network [54–56]. This can be achieved using a biopolymer scaffold, in order to control the delivery of cells, growth factors, or drugs [57]. This biopolymer scaffold will also limit cell dispersion within the cavity, and preserve them from the hostile environment and produces the ideal microenvironment for cells to prosper [58]. The 3D architecture and structure for brain tissues have been reviewed recently [54] with parameters such as design factors, fabrication methods, and mechanical properties key to achieve tissue constructs that mimic brain-specific architecture. In addition, hydrogels are the most commonly deployed materials to recreate the brain architecture. It has been demonstrated that some hydrogels like HA, alginate, chitosan (Cht), methylcellulose, fibrin, collagen type I, and self-assembling peptides are best suited to meet the requirements for brain injury therapies [59].

#### Key scaffold properties for brain tissue regeneration

The in vivo biodegradability is highly important*,* as it avoids secondary surgery to remove the scaffold from the brain. For example, many studies have been conducted to control the crosslinking and hence degradation of HA-based films and scaffolds for tissue engineering purposes [60–65]. Furthermore, HA-based scaffolds provide intrinsic immunomodulatory and antibacterial properties enhancing the performance of tissue-engineered constructs [66, 67].

**Fig. 1:**
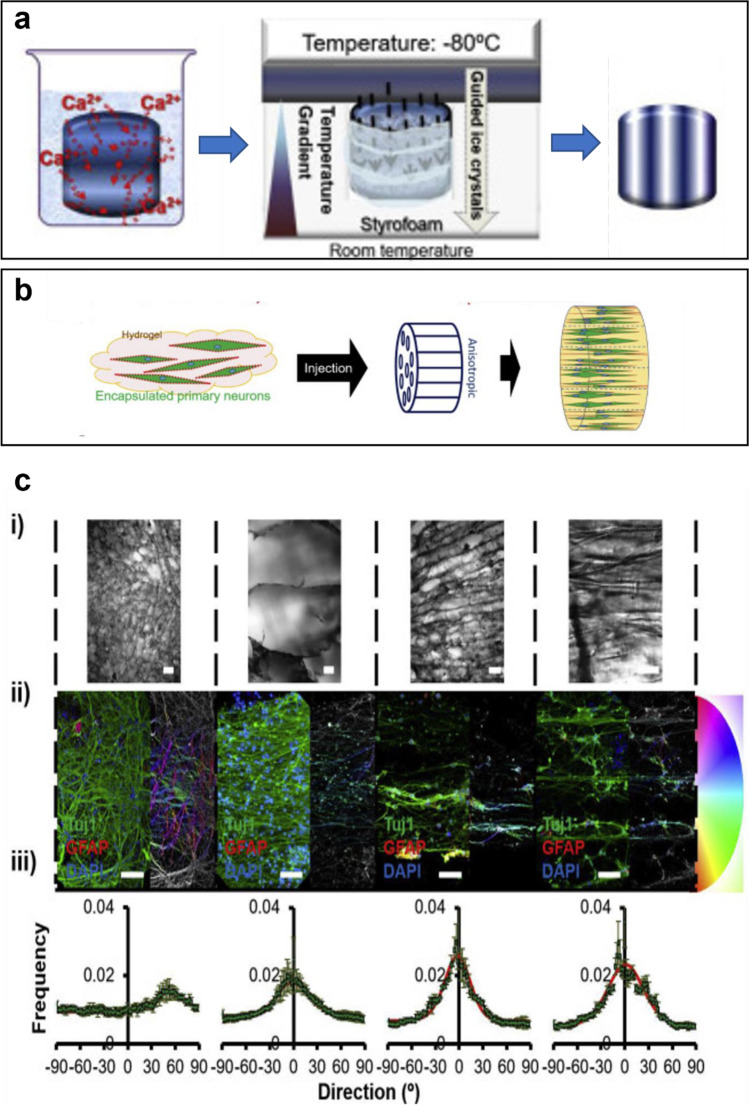
**a** Production of anisotropic 3D porous scaffolds based on gelatin methacryloyl-anhydride modified gellan gum (GelMA-MAGG). **b** Schematic of anisotropic porosity guiding neurite outgrowth from mice primary neurons. **c** Anisotropic 3D porous structures. (i) Bright-field images for porosity determination (scale bar = 50 μm). (ii) Primary neurons cultured in the 3D oriented porous structures to evaluate the neurite outgrowth guidance ability. The colour map represents the orientation angle of neurites. (iii) Cell distribution and orientation analysis and quantification. A fitting curve was traced based on a Gaussian function of the frequency of orientation angle events. Reproduced with permission [79]. 2018, Elsevier Ltd

The degree of porosity and pore size also dictates degradation rates of scaffolds suitable for many types of tissue, including brain tissue regeneration [68–71]. The brain itself contains approximately 80% water [72] and is composed of immune cells and enzymes. Thus, depending on the implant site within the brain, biomaterials may undergo hydrolytic or enzymatic degradation [73]. In order to improve the stability of the implants, different crosslinking approaches have been reported in the literature for the production of scaffolds for brain tissue regeneration, including physical crosslinking, photo-crosslinking, interpenetrating polymer networks, and enzymatic crosslinking [74–77]. Aligned electrospun poly(lactic-co-glycolic acid) (PLGA) scaffolds functionalised with hydrolysed monosialotetrahexosylganglioside (LysoGM1), a prominent brain ganglioside, have been developed to boost the endogenous regeneration providing significant benefits for reconstruction of neural tissues with the LysoGM1 still preserved after hydrolysis [78]. LysoGM1 enhanced the scaffold hydrophilicity allowing faster degradation. Canadas et al. [79] fabricated anisotropic 3D structures made of methacrylated gellan gum (MAGG) and gelatin (GelMA), shown in Fig. 1a and b. They controlled the ratio of GelMA:MAGG to tune the mechanical and structural properties such as the degradation rate, elastic modulus, and pore size of the resulting scaffolds, allowing modulation of neurite extension and orientation in vitro. Primary cortical neurons were isolated from embryonic day 18 (E18) C57bl/6 mice, and cultured onto anisotropic scaffolds, as shown in Fig. 1c. They studied the effect of pore size on the orientation of neurite outgrowth. Pores ranged from 20 to 400 μm, with the growth of neurites following a random orientation due to anisotropy. Only when the pores are ordered, the growth of neurites is oriented. The layers of neurites outgrowth had different diameters according to the diameter of the pores, and the direction of neurite outgrowth is determined as shown in Fig. 1.

Scaffolds may also exhibit mechanical and topographical cues through the use of nanofibers [80], or even further influence cell behaviour by their potential conductive ability [81]. Vaysse et al. [82] implanted grooved micro-patterned polydimethylsiloxane (PDMS) scaffolds in a rat model and demonstrated that the guidance of pre-seeded neuronal cells is promoted along micro-channels. This scaffold enhanced recovery of the rat motor functions and the implant appeared not to have induced inflammatory response. The same scaffold was re-employed 2 years later with NSCs and it promoted tissue reconstruction through survival conditions improvement for both endogenous neural cells and grafted NSCs [83]. Swellability of a scaffold (especially hydrogel-based scaffolds) is also advantageous in terms of permeability, flexibility, and biocompatibility [54].

**Fig. 2:**
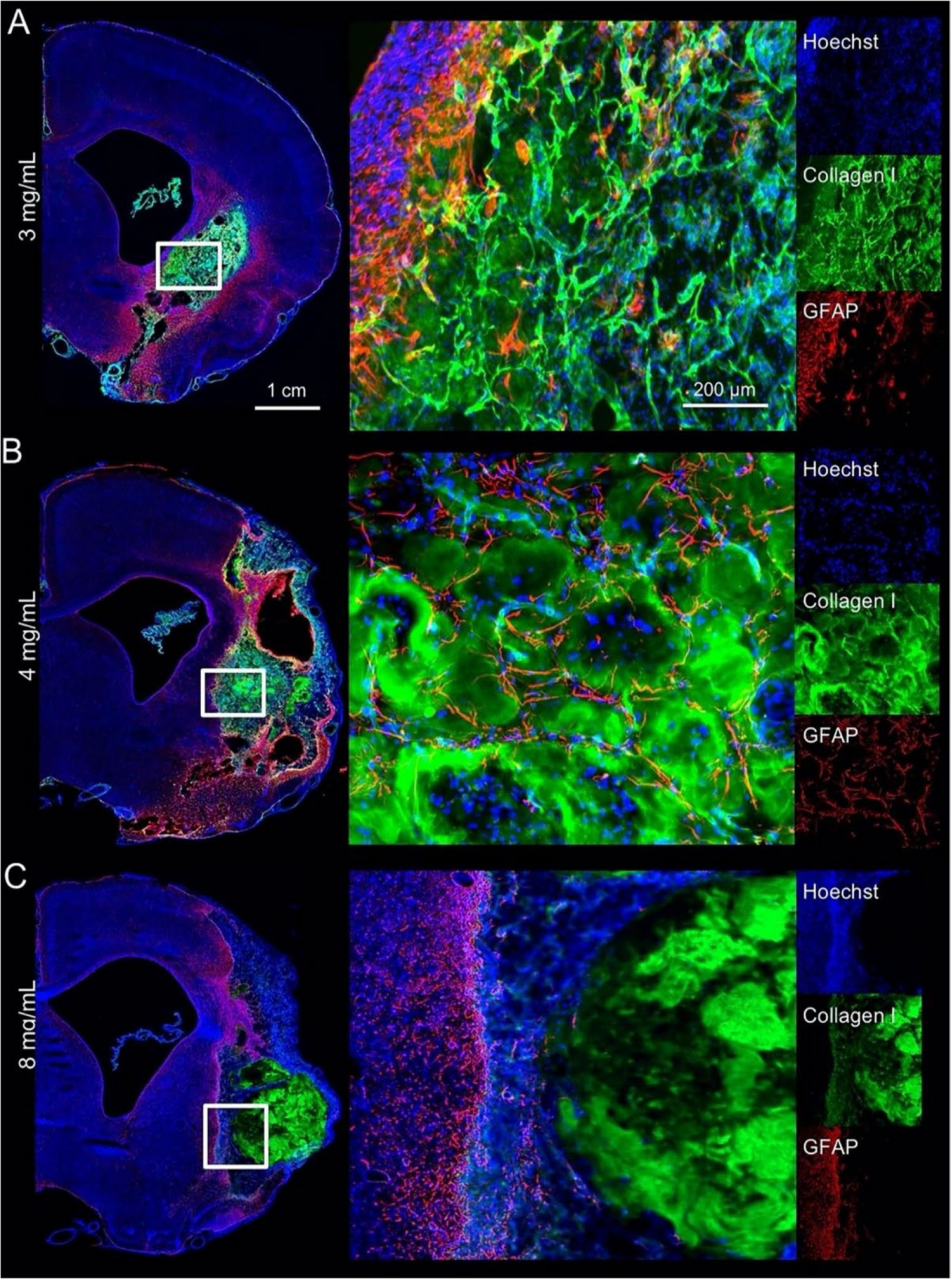
**A** The distribution of the invading GFAP + cells seen throughout the remaining hydrogel is even. **B** GFAP + chain cells invasion filling the space in between patches of ECM hydrogel as identified by collagen I staining can be seen. **C** Lower density of cells in the hydrogel at 90 days compared to the less concentrated gels. Reproduced with permission [89]. 2018, Elsevier Ltd

The overall aim of using scaffolds, which can be composed of synthetic or natural polymers, is to mimic the ECM brain features. In terms of mechanical properties, scaffolds should exert an elastic modulus lower than 1 kPa [84]. As they are surgically implanted, the architecture and structural integrity of the scaffold can be damaged during surgery. The advent of injectable hydrogel systems has largely overcome this as they can be injected directly into the brain cavity while modulating its stiffness [59, 85–88]. Ghuman et al. [89] injected varying concentrations of an ECM-based hydrogel in a rat model for 90 days. Results showed that when the concentration is low, in their case 3 mg·ml^−1^, the efficiency of the hydrogel in terms of cavity progression and volume reduction was higher. Also, cell infiltration was improved, as shown in Fig. 2. Macrophage density affects biodegradation with fast biodegradation within 14 days [89]. On the contrary, another study authored by Zhang et al. [87] demonstrated that decreased degradation rates are linked to higher cell densities. They loaded and injected human umbilical cord mesenchymal stem cells (hUC-MSCs) within a composite hydrogel, composed of HA and sodium alginate (SA). SA provided mechanical support for cell growth, whereas HA regulated cell adhesion, neuronal migration, and neurite outgrowth [87]. This scaffold contributed to the regeneration of endogenous nerve cells. This suggests that cellular behaviour is dependent on the environment. Therefore, as these results are contradictory, further studies need to be conducted.

Brain damage is associated with spatial imbalance of cholinergic system; therefore, maintenance of cholinergic system is extremely important. An injectable hydrogel consisting of acetylcholine-functionalised graphene oxide and poly(acrylic acid) promotes neurite outgrowth, stabilises microtubule networks, and enhances the expression of some key neural markers in rat cortical primary neurons. Furthermore, this hydrogel exhibits significant potential in neuroregeneration and also promotes fast recovery of the sham-injured mice brain through local release of acetylcholine in the injured brain [90].

Implanting exogenous stem cells for the formation of new tissues implies two strategies: either using mature neurons that exhibit a suitable phenotype but might not all survive the transplantation [91] or either using undifferentiated cells which might survive the transplantation but upon which we have no control of their differentiation [92]. One study performed by Payne et al. [93] suggested with an experiment in vitro that the difference in maturity would affect the cells survival ability. They also performed experiments in vivo to investigate the influence of cell maturity on neural progenitor cell survival [94]. They evaluated three models: (i) an early-differentiated model, 0 days in vitro, (ii) a mid-differentiated model, 16 days in vitro, and (iii) a late-differentiated model, 32 days in vitro, within a rat stroke model. The experiment required a hyaluronan and methylcellulose (HAMC) hydrogel carrier and they discovered four interesting points. (i) Enhanced recovery performance using HAMC associated with early- and mid-differentiated cells for 56 days was observed. However, results with late-differentiated cells were not conclusive. (ii) No significant difference in cells survival and proliferation between the three models was observed. (iii) It demonstrated the link between cells death/loss of mature phenotype and cells detachment and injection. Early-differentiated cells did not significantly change during transplantation. (iv) Finally, late-differentiated cells induced an increase in host injury and gliosis, causing greater damages. Thus, the carrier and cell maturity are important for brain tissue recovery. Others similarly found no difference in cell survival depending on maturity [95, 96]. However, another study showed that pre-differentiated NSC fostered endogenous neurogenesis whereas undifferentiated cells developed into astrocytes, contributing to the glial scar tissue formation [91]. These results suggest that finding the balance of mature and immature cells is tricky to identify.

#### Scaffolds combined with growth factors

The presence of neurotrophic factors is essential for neuronal cell differentiation and maturation, as they modulate the expression of enzymes for neurotransmission, dictating neuronal phenotype [97]. Neuronal migration, proliferation, survival, and differentiation can be improved with the addition of exogenous biological factors [59]. Their delivery to the injured site of the CNS can prevent massive degeneration and help to re-establish the neuronal network. Several GFs have been demonstrated to have a positive effect on brain injury repair. Within them, the VEGF, EGF, brain-derived neurotrophic factor (BDNF), EPO, nerve growth factor (NGF), basic fibroblast growth factor (bFGF), and neurotrophin-3 (NT-3) have gained great attention within the scientific community as they are known to play an important role in neurogenesis regulation [97].

However, the direct delivery of neurotrophic factors to the injured site in the brain has been shown to be inefficient, displaying poor infiltration through the BBB. The BBB is composed of ECs basement membrane, astrocytic foot processes, and pericytes [22]. The delivery and action of the GFs gets improved when they are administered using a scaffold [98–100]. Therefore, various biomaterial-based strategies are being developed to administer drugs such as BDNF protein to the injury site [101, 102]. BDNF-loaded PLGA NPs display good penetration of BDNF resulting in improvement of neurological and cognitive deficits after TBI. NPs are transported across the BBB either via endocytosis by the ECs or by transcytosis across the endothelium, with subsequent release of the drug within the cells and therefore into the brain [103]. A block copolymer of poly(ethylene glycol) and poly(lactic acid) (PEG-PLA) NPs delivered BDNF successfully also, enhancing BBB penetration and improving memory/cognition in a mouse model post-stroke [104].

It is also necessary to deliver GFs via a localised and efficient method to achieve good therapeutic effect. A collagen-binding BDNF was injected into the lateral ventricle of a rat model [105, 106]. The role of the collagen was to specifically target this area given that the ventricular ependyma of the brain is rich in collagen and this injection enhanced endogenous cell proliferation in the SVZ. The activation of endogenous NSCs from the SVZ is a promising strategy for neuroregeneration [107]. Therefore, Jyan et al. [108] studied the injection of a HA hydrogel containing sulphated glycosaminoglycan-based NPs made of HS and CS to mimic the brain ECM and control the delivery of stromal-derived factor-1α (SDF-1α) and bFGF. A novel delivery system capable of circumventing the blood–brain barrier and directly releasing growth factors to the brain in a sequential manner is key for tissue repair. Pegylated epidermal growth factor (EGF) (EGF-PEG) encapsulated in PLGA nanoparticles and EPO in biphasic microparticles comprised of a PLGA core with a poly(sebacic acid) coating were dispersed in a HAMC hydrogel which spatially confines the particles and attenuates the inflammatory response of brain tissue. It mediated sequential delivery of EGF-PEG and EPO leading to tissue repair in a mouse stroke model with minimal damage compared to ICV infusion. This method of drug-loaded scaffolds is promising as it avoids invasive approaches. Logan et al. [109] found similar results when delivering bFGF and NT-3 sequentially. Epicortically placed, EGF-PEG was employed with a HAMC hydrogel and showed increased NSC proliferation in a mouse model [110]. However, this needs to be repeated in human brain where the distance between the epicortical and subcortical structure is larger [111].

**Fig. 3:**
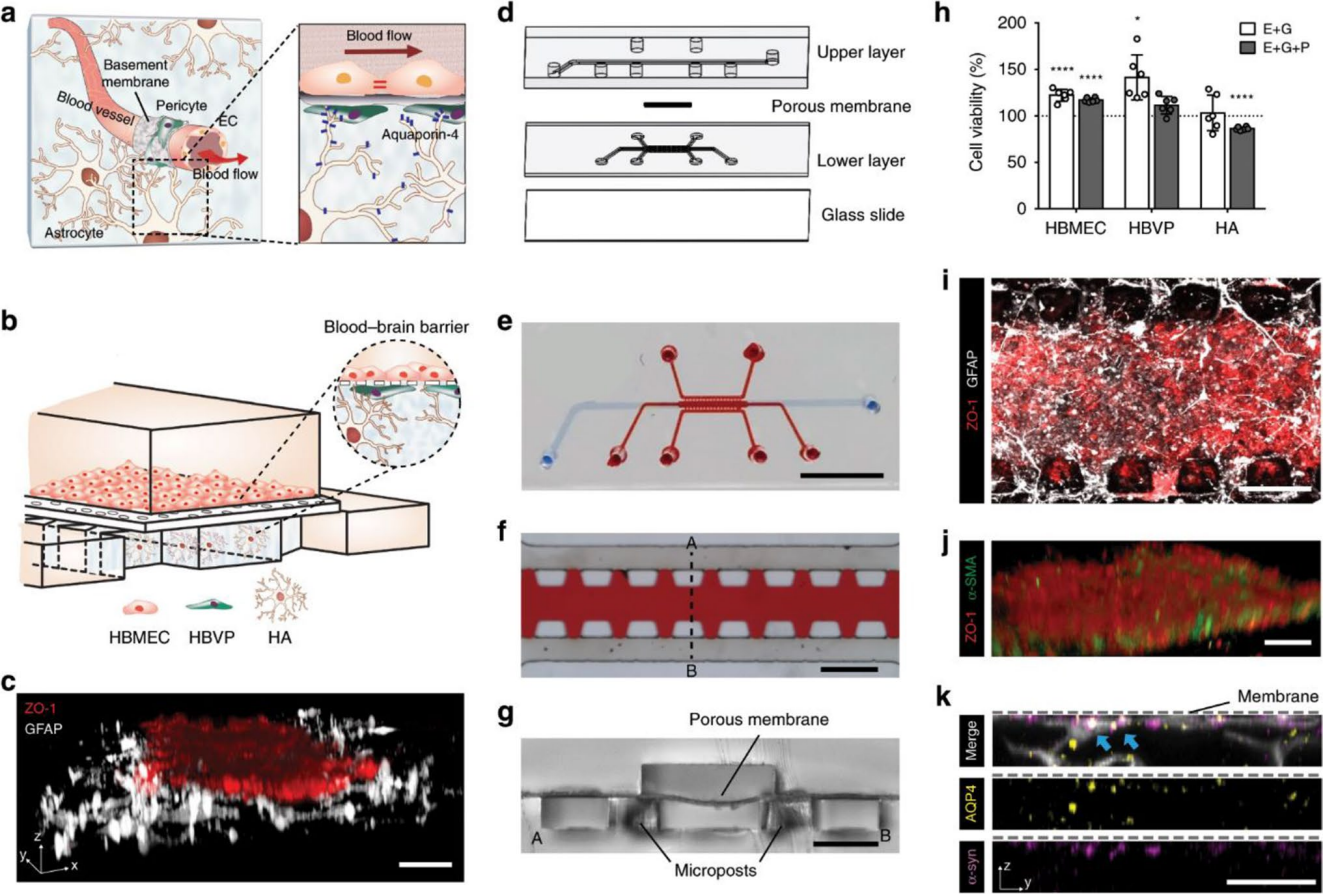
**a** Schematic description of the BBB consisting of endothelial cells (ECs) along the blood vessel under continuous blood flow, pericytes covering the endothelial monolayer, and astrocytes with aquaporin-4 (AQP4) expression at their end-feet near the blood vessel. **b** Schematic description of our microengineered human BBB model. **c** 3D configuration of the BBB model showing human brain microvascular endothelial cells (HBMECs) (ZO-1, red) and human astrocytes (HAs) (GFAP, white) (scale bar = 100 µm). **d** Explosion view of the device consisting of upper vascular layer, porous membrane, lower perivascular layer, and glass slide. **e** A photo of the device after completing fabrication of the device (blue: upper channel and red: lower channels) (scale bar = 500 µm). **f** Lower layer consisting of three parallel channels separated by series of micropillars (red: centre channel) (scale bar = 500 µm). **g** Cross-section of the device after fabrication (scale bar = 200 µm). **h** Cell metabolic activities assessed by a (3-(4,5-dimethylthiazol-2-yl)-5-(3-carboxymethoxyphenyl)-2-(4-sulfophenyl)-2H-tetrazolium) (MTS) assay (E + G: 1:1:1 mixture of endothelial medium, astrocyte medium, and microglia medium, E + G + P: 1:1:1:1 mixture of endothelial medium, astrocyte medium, microglia medium, and pericyte medium) (data represent mean ± s.d. of *n* = 6 for each condition, *p < 0.05 and ****p < 0.001 versus each cell culture medium by Student’s *t*-test). **i** Bottom view of the device with endothelial monolayer (ZO-1, red) and astrocytic network (GFAP, white) (scale bar = 50 µm). **j** Endothelial monolayer (ZO-1, red) supported by a layer of human brain vascular pericytes (HBVPs) (α-SMA, green) (scale bar = 50 µm). **k** Aquaporin-4 (AQP4, yellow) and α-syntrophin (α-syn, magenta) expressions at astrocytic end-feet (GFAP, white) underneath a porous membrane (indicated as the dotted line) in the lower channel (blue arrows indicate co-localisation of AQP4 with α-syn.) (scale bar = 50 µm). All images are representative ones from at least five biological and three technical replicates [113]

This difference highlights that while a number of strategies developed to successfully pass therapeutic treatments such as NPs through the BBB have been reported, another challenge arises in the form of evaluating the effectiveness of these strategies in human models. Often, evaluation techniques which focused on cell interaction or even animal models show poor translation into human treatments due to species differences [112]. Therefore, the development of an in vitro model of the human BBB, as recently reported by Ahn et al., will allow for an alternative way to investigate not only the effectiveness of NPs to penetrate the BBB by means of direct quantitative analysis but will also allow for this investigation to be conducted in real time. The development of the model is shown in Fig. 3 [113]. The BBB chip model was developed to mimic the BBB in its function and structure by utilising a monolayer of brain endothelial cells, as well as a 3D network of astrocytes with polarised expression of aquaporin 4 (AQP4) and reduced reactive gliosis.

HA hydrogels have delivered BDNF in mice and monkey models promoting neurogenesis in peri-infarct tissues with improved motor function recovery [114]. Approaches using HA gels or sulphate proteoglycans have been extensively employed [115] as they mimic brain ECM. Micropillared PCL was used to deliver BDNF in vitro [116]. Neuronal survival is promoted during the early and critical phases of adhesion and network development. Furthermore, BDNF is released, enabling neuronal adhesion. It was 30% more efficient compared with the initially available BNDF quantity, with biodegradability enhanced by the high surface/volume ratio of the scaffold leading to a quicker early release of BDNF. NT-3, combined with chitosan, engaged endogenous NSCs to migrate and proliferate, restoring brain functions [117, 118]. Finally, NT-3 improved the outcome after TBI by modulating the environment and thus exhibiting anti-inflammatory and neuroprotective actions.

**Fig. 4:**
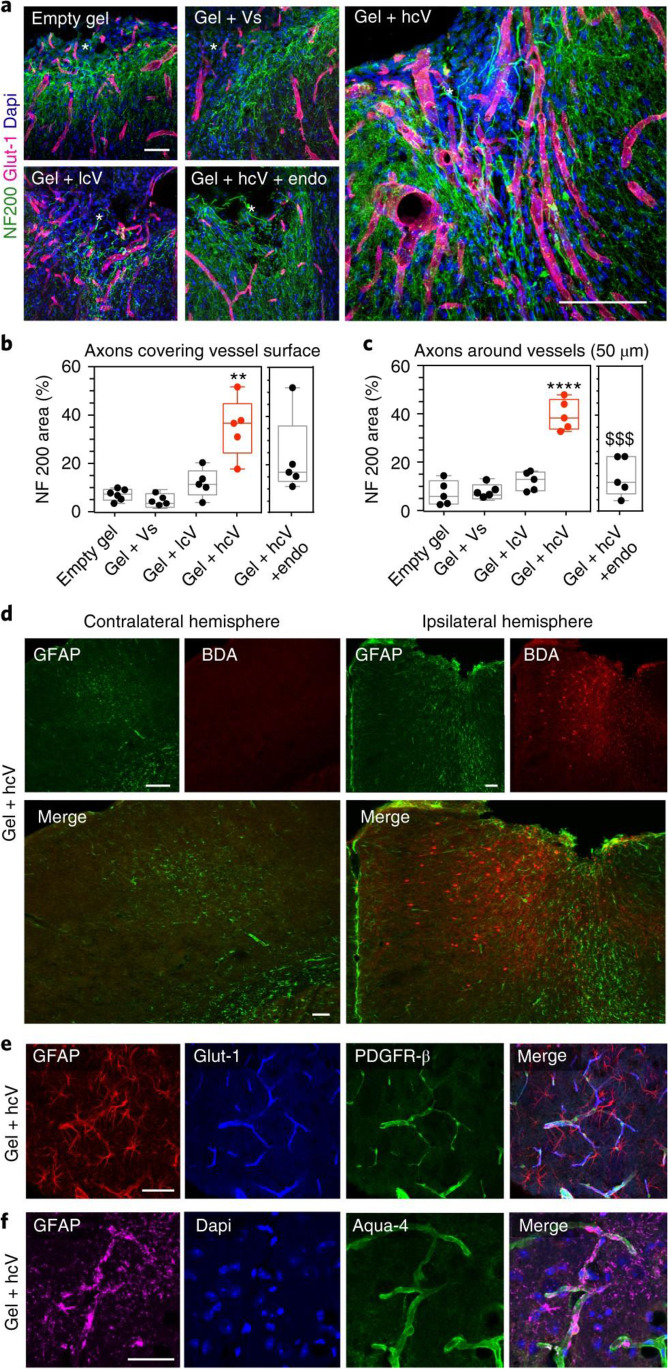
**a** Fluorescence microscopy images of vessels (Glut-1 (red)) and axonal neurofilaments (NF200 (green)) in and around the stroke site (*) 16 weeks after gel transplantation. **b** Quantitative assessment of the proximity between the two networks with the quantification of NF200-positive signal on vessels and **c** NF200-positive area a distance of 50 µm from vessels. **d** Fluorescent images of the peri-infarct astrocytic scar (GFAP (green)) and BDA-traced neurons (red) in the ipsilateral hemisphere of gel + hcV-injected mice 16 weeks after gel transplantation. **e** Fluorescent images of astrocytes (GFAP) co-stained with vessels (Glut-1) and pericytes/smooth muscle cells (PDGFR-β) or **f** with astrocyte end-feet (aqua-4) in the stroke site of hcV-treated mice 16 weeks after gel transplantation. Empty gel, HA hydrogel (gel); Vs, 200 ng of Vs; lcV, 1 µg of nH loaded with 200 ng of VEGF; hcV, 0.01 µg of nH loaded with 200 ng of VEGF and 0.99 µg of unloaded nH; endo, a daily i.p. injection of endostatin on days 5 to 15. Data are presented using a minimum-to-maximum box plot. Each dot in the plots represents one animal and *p* values were determined by one-way ANOVA with a Tukey’s post hoc test. **p < 0.01, *p* < 0.0001. Data represent the average. *p* < 0.001 versus gel + hcV. Scale bars, 100 µm. Reproduced with permission [119]. 2018, Springer Nature

**Angiogenesis specific growth factors*:*** The creation of a new vascular network is required for cellular migration within the brain cavity since blood vessels transport oxygen and nutrients. Vascularisation is commonly promoted using EGF or vascular endothelial growth factor (VEGF) with the use of varying biomaterial scaffolds [55], given that VEGF only delivery to the brain has previously been associated with immature blood vessels [119]. Scaffold-based delivery systems have been shown to be efficient in fostering vascularisation. For example, a nanofibrous self-assembling peptide hydrogel loaded with VEGF created a strong vascular network at day 7 and day 14 post treatment, increasing cell infiltration, repairing the BBB, and reducing coagulation [85]. Similarly, another study showed enhanced vascularisation at 2 and 16 weeks with injectable HA hydrogel systems with high clustered VEGF condition in a mouse stroke cavity model, when injected 5 days post-stroke. High clustering of VEGF (hcV) combined with the HA hydrogel increased the proliferation and cell area coverage of endothelial and pericyte cells in and around the stroke cavity area, when compared to the other groups. A high association between the vessel and axonal network formation was also found at 16 weeks, and is depicted in Fig. 4a–c, with the vessels stained by means of Glut-1 and axons with NF200. Blocking angiogenesis in the hcV group reduced the axonal network, especially around the vessel area. This highlights the importance of the presence of vessel structures to allow for axonogenesis to occur. The maturity of the vessels was shown to contain normal brain vasculature by means of GFAP and AQP4 staining of astrocyte and astrocytic end-feet, as well as pericytes by means of platelet-derived growth factor receptor beta (PDGFR-β) staining, as depicted in Fig. 4e–f [119]. In a separate study on the role of VEGF in the nervous system, increased angiogenesis was followed by an increase in inflammation VEGF and was shown to enhance motor and sensory functions in a rabbit model of ischemic peripheral neuropathy [120]. Application of VEGF after stroke injury can decrease brain infarct size, via promotion of angiogenesis and neurogenesis. In further support of a role for VEGF in damage control during brain injuries, application of VEGF to the contused spinal cord produced behavioural and cellular improvements [121, 122], and VEGF significantly enhanced nerve regeneration when applied to Matrigel implants in injured sciatic nerves [123].

However, other studies have shown that VEGFs loaded on biomaterials were not suitable 6 weeks post-injury [124, 125]. RADA16, self-assembled peptide, when conjugated with SVVYGLR, demonstrated good results in zebrafish [126]. SVVYGLR, which is the functional motif in osteopontin molecules [127], allowed ECs to form a vascular structure as well as their migration and adhesion. Angiogenesis enabled better neurogenesis and thus enhanced zebrafish optomotor response over 28 days.

Blood vasculature can act as physical support providing adhesion-dependent signals for migration, proliferation, differentiation, and survival of cells, especially neuroblasts [128]. Under some pathological conditions such as a stroke, neuroblasts are upregulated from the SVZ and from the SGZ [129] to repopulate the cavity. Their individual migration is known to display poor directionality [130], suggesting that guidance cues and specific bindings are needed. For example, β1 integrin is necessary for their efficient migration along blood vessels [131]. Neuroblast migration has shown to be more dependent on astrocytes since astrocytic tunnels called glial tubes surround neuroblasts, rather than blood vessels themselves [132]. An interesting study fabricated an astrocytic network coated with collagen I within an agarose hydrogel to facilitate neuroblasts migration [133]. They provided structural cues to enable neurite extension through the glial scar tissue directly along the aligned astrocyte network, but it required a specific column diameter and high seeding density of astrocytes. Instead of seeding astrocytes during the scaffold preparation, a study implanted a PCL microfiber coated with self-assembled colloidal graphene near the SVZ in a rat model and showed astrocytic growth processes within the scaffold. It enabled the redirection of neuroblasts from the SVZ along the implant to potential target regions of the brain [134]. They also found that the graphene reduces microglia activation and macrophage infiltration, stopping them at the outermost scaffold layer until the third week following implantation and thus reducing inflammatory progression. After the seventh week, both the ingrowth depth and process density declined. The behaviour of astrocytes depended on surface of the scaffold, where adhesive layers of poly-L-lysine were more suitable than non-adhesive heparin layers. This work highlights the importance of the modulation of the inflammatory response as a strategy to improve brain tissue regeneration.

### Strategies to combat glial scar tissue

In a recent review, likely candidates for clinical trials are discussed along with current knowledge of the scar-modulating treatments, it concludes that a combinatorial strategy is likely to help eliminate the detrimental effects of scar tissue on CNS repair [135].

Although the prevention of glial scar tissue formation can prevent damage expansion through a thick network of reactive astrocytes and macrophages, without glial scar tissue axonal regeneration and cell migration is difficult. During inflammation, the glial scar tissue releases cytokines, such as interferon-γ, IL-1, IL-2, IL-6, TNF-α, and macrophage stimulating factors [136], and the modulation of this scar tissue formation is an interesting approach to circumvent inflammation. Engineered nanoparticles (NPs) of heparin were utilised to produce an immune-modulating angiogenic biomaterial capable of direct delivery to the stroke cavity to promote tissue formation de novo, resulting in axonal networks along regenerated blood vessels. This allowed functional recovery in tissue through established axonal networks. The approach generates a vascularised network of regenerated functional neuronal connections within previously dead tissue and lays the groundwork for the use of angiogenic materials to repair other neurologically diseased tissues [119]. Another promising strategy to overcome scar tissue formation is grafting scaffolds that can disturb the scar blockade, enabling cell invasion into the wound. An aragonite skeleton of corals has been used as a scaffold for testing this strategy, and its effect can be regulated by engineering the scaffold’s surface topology. It was found that grafting coralline scaffolds of predesigned surface roughness and porosity into brain wounds, control over scar tissue formation could be achieved, providing an opportunity for cell migration and damage repair [137]. Two proteins and one proteoglycan found in central nervous system extracellular matrix, as well as fibrinogen, were patterned in stripes onto collagen hydrogel and astrocytes were cultured on these surfaces. This created astrocyte layers in which cells were aligned with underlying patterns and had reduced chondroitin sulphate expression compared to the cells grown on collagen alone [138].

Astrocytes are a target for regenerative neurobiology because in brain injury models, their phenotype arbitrates brain integrity, neuronal death, and subsequent repair and reconstruction. A recent study illustrates the therapeutic potential of bioengineering strategies using 3D electrospun scaffolds which direct astrocytes into phenotypes supporting brain repair. Findings demonstrate murine astrocytes adopt a healthy phenotype when cultured in 3D. Astrocytes proliferate and extend into poly-ε-caprolactone scaffolds displaying 3D stellated morphologies. These scaffolds have potential to direct inflammation in such a way as to aid regenerative neurobiology [107].

### Cells-based strategies

Cell therapy involved in brain regeneration includes various undifferentiated cells such as neuronal stem cells (NSCs), neural progenitor cells, and neural precursor cells (NPCs). NSCs have the ability to self-renew and the potential to differentiate into neurons and glial cells [139], compared to neural progenitor cells that are the progeny of NSCs, which exhibit no self-renewal and do not generate the non-neural cells that are also present in the CNS, such as cells from the immune system [140]. Regarding NPCs, derived from ESCs or iPSCs, they consist of undifferentiated progenitors of NSCs [141]. Derivation of iPSCs for NPCs should be employed to circumvent ethical issues associated with ESCs as they imply the destruction of a human embryo [142].

The combined use of cells with biomaterials, such as hydrogels or biodegradable scaffolds, greatly increases the viability of exogenous cells and, consequently, functional recovery probabilities [143]. For example, a HA hydrogel containing PLGA NPs promoted NSCs survival and growth in vitro, through the continuous delivery of BDNF and VEGF [144]. Moreover, CS glycosaminoglycan (CS-GAG) showed to support NSCs in vitro and transplanted cells in vivo following TBI. CS-GAG promoted bFGF retention and promoted the maintenance of the encapsulated undifferentiated NSCs. Neural progenitor cells derived from iPSCs were encapsulated in a bFGF-binded CS construct [145]. Their transplantation promoted sensorimotor behavioural outcomes in a mouse model.

The potential of human umbilical cord-derived mesenchymal stem cells (hUC-MSCs) has been demonstrated in several studies [145–148], as they present excellent proliferative ability, low immunogenicity, and are an easily sourced [149, 150]. Shi et al. [148] performed a study focused on the effect of BDNF on hUC-MSCs differentiation in vitro. They immobilised BDNF on chitosan hydrogel scaffolds cross-linked with genipin [151]. They provided a continuous release of BDNF for 30 days [148]. In a study in vivo, the same team transplanted hUC-MSCs into the cavity of a rat model, showing an improved tissue regeneration after TBI [152]. They used the same scaffold with BDNF to focus on the SDF-1α/CXCR4 application [143]. The overexpression of CXCR4 from hUC-MSCs in response to SDF-1α from the scaffold demonstrated excellent cell migration behaviour and the BDNF release improved cell differentiation. Not only had this type of scaffold enhanced hUC-MSCs therapeutic strategy but it also protected loaded cells within. A sericin-based hydrogel [153], cross-linked via genipin, was also found to be protective towards loaded cells and primary neurons induced after stroke [154].

Peptide addition to biomaterials tends to help anchorage or provide adhesion mechanisms for neurons. For instance, an injectable 3D silk fibroin (SF)-based hydrogel scaffold with encapsulated NSCs was developed by Sun et al. [155]. It showed enhanced cell viability and neuronal differentiation. The bioactivity of SF with NSCs was improved using an IKVAV peptide. The IKVAV peptide is a short laminin peptide which has been demonstrated to facilitate cell anchorage and adhesion, neurite outgrowth, and angiogenesis, probably due to its presence in the ECM [156, 157]. This peptide sequence was linked to a self-assembled peptide hydrogel (SAPH) and implanted in a rat model [158]. The adhesion and neuronal differentiation of the encapsulated NSCs occurred due to the presence of the IKVAV peptide. An enhanced survival of the encapsulated NSCs and a reduction of glial astrocytes formation was noticed, whereas the transplantation of NSCs without the presence of the peptide resulted in a limited survival rate and poor regeneration. IKVAV peptide is usually conjugated with others such as RADA16-I, with good outcomes [157, 159]. This association circumvents two main drawbacks: (i) IKVAV alone is not able to self-assemble into a hydrogel [159] and RADA16-I peptide is known to be detrimental for cell viability, related to low pH [160].

**Fig. 5:**
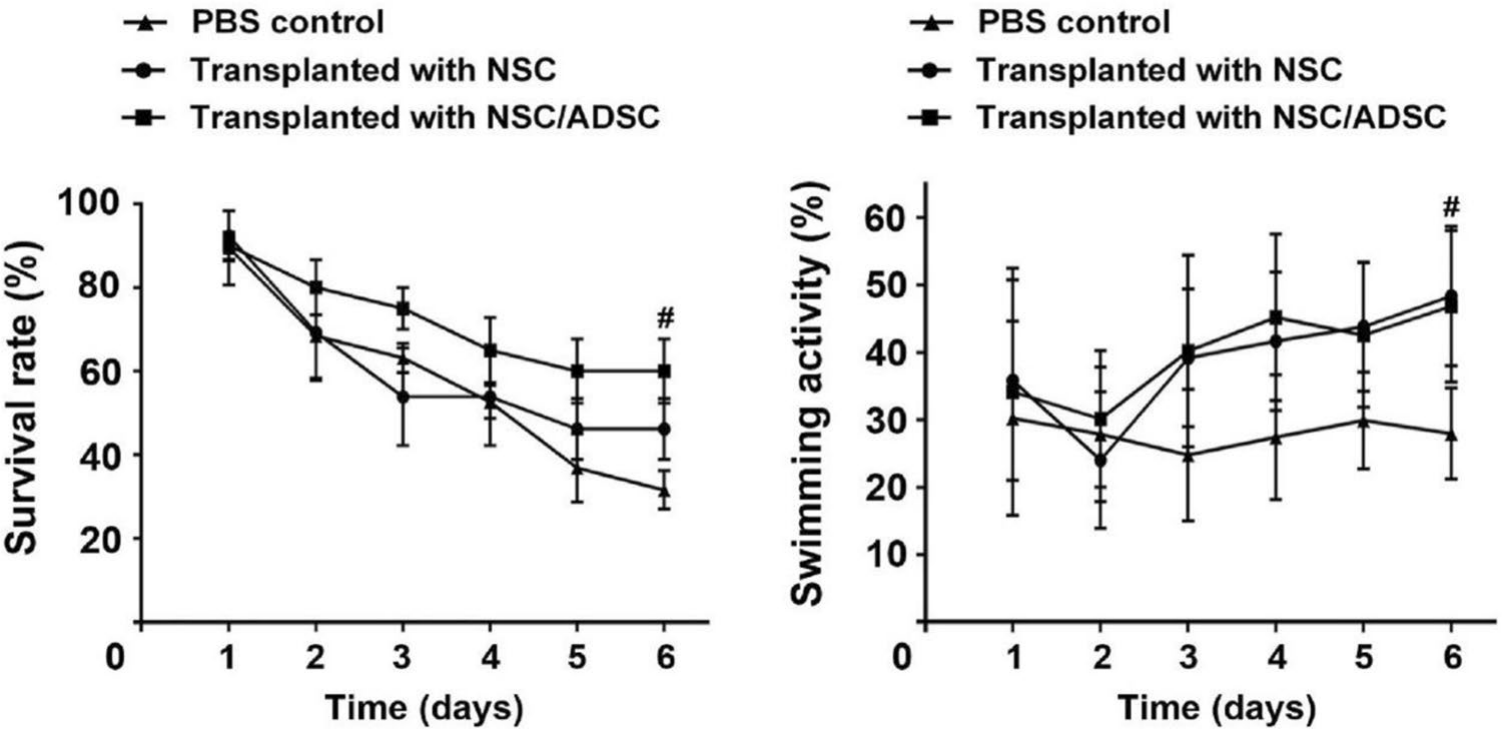
The survival rate and swimming activity of injured adult zebrafish from 1 to 6 days after cell transplantation. Reproduced with permission from [167]. 2017, Elsevier Ltd

Given the low survival rate of NSCs after engraftment [161], biomaterials such as HA hydrogels are used, which have been proved to improve NSCs survival and proliferation [162]. Another approach is focused on spheroid stem cell culturing. Compared to monolayer cell culture, it has been shown to facilitate cell/cell and cell/matrix interactions [163] and it has been applied as a treatment for TBI/stroke injury [164, 165]. An in vitro study demonstrated that spheroids made of chitosan for NSCs culturing inferred the cells with an enhanced self-renewing capacity and plasticity [166]. Further experiments in vivo demonstrated in a zebrafish model the positive outcomes when the chitosan spheroids are used for NSCs and MSCs culture [167]. The spheroid shell contained NSCs and at its core MSCs. The transplantation of NSCs/MSCs increased NSCs survival rate and its swimming activity which is presented in Fig. 5. In a more recent work by Han et al. [168], an in vitro experiment demonstrated the formation of a neurovascular network when NCSs and ECs were injected in chitosan-HA hydrogel spheroids.

Regarding stem cell culturing, the use of organoids is gaining interest for brain tissue regeneration applications as in vitro studies highlighted brain features recapitulation, such as brain expression markers, and enhanced cell maturation [169–171]. Three in vivo studies transplanted cerebral organoids after stroke promoting exogenous cell growth at the peri-infarct site and endogenous cells from the SVZ [172–174]. In the three studies, organoids vascularisation, neuro-differentiation, and cell survival were demonstrated. In the most recent one, the transplantation reduced the cavity volume, and improved rat motor functions as well as synaptic reconstruction [172]. Identifying the correct time window for transplantation is a critical parameter for potential success, and in the case of cerebral organoids, it was associated with a time window of 6 h post-stroke.

Table 2 highlights that even if tissue regeneration occurs, motor function recovery does not always happen. Moreover, results differ depending on studies because animal models possess differing anatomies, suggesting that those studies might not be relevant to humans. Moreover, combinations of scaffolds, cells, and GFs are quite heterogeneous, which render the analysis more difficult. However, it can be noted that the combination of scaffold/GFs enabled the migration, proliferation, and differentiation of exogenous cells. Inflammatory modulation, as well as tissue protection, is also improved when GFs are incorporated. The addition of cells also improves tissue regeneration and recovery. However, it is not clear yet how to best combine cells and GFs, and in which scaffold type. FDA-approved thrombolytic therapies have also shown good outcomes for ischemic stroke but it must be administered within 3 or 4.5 h following stroke [48, 49].

**Table 2:** In vivo studies of scaffolds combined with cells/growth factors [68, 70, 77, 87–90, 93, 94, 96, 100, 126, 133, 156]

Scaffold	Cells	Growth factor	Disease	Animal model	Effects	Ref
SAPH		VEGF	TBI	Rat	Wound recovery	[85]
HA and SA hydrogel	hUC-MSC		TBI	Rat	Motor and memory recovery	[87]
HAMC hydrogel	NPC		Stroke	Rat	Growth and survival highlighted	[94]
PLGA NPs		BDNF	TBI	Mice	Neurological and cognitive improvements	[103]
PEG-PLA NPs		BDNF	Ischemic stroke	Mice	Behavioural recovery	[104]
Collagen		BDNF	Ischemic stroke	Rat	Neuronal regeneration and protection	[105]
HA hydrogel & GAG NPs		bFGF	Ischemic stroke	Rat	Improved neurogenesis and angiogenesis	[108]
HAMC hydrogel		EGF-EPO	Ischemic stroke	Mice	Reduced inflammatory response and tissue repaired	[175]
HAMC hydrogel		EGF	Stroke	Mice	Better response of NSPCs	[110]
HA hydrogel		BDNF	Stroke	Mice/monkey	Motor functions recovered	[114]
Cht		NT-3	TBI	Rat	Neural repairs	[118]
CS	NPC	bFGF	Stroke	Mice	Sensorimotor functions improvements	[145]
Cht hydrogel	hUC-MSC	BDNF	TBI	Rat	Tissue regeneration	[152]
SAPH + IKVAV peptide	NSC		TBI	Rat	Tissue regeneration	[158]
Cht hydrogel	MSC/NSC		Stroke	Zebrafish	Motor functions recovery	[167]
HA hydrogel		VEGF	Stroke	Mice	Functional recovery	[119]
SAP	EC		Stroke	Zebrafish	Optomotor response	[114]

#### Cell signalling pathways

**Fig. 6:**
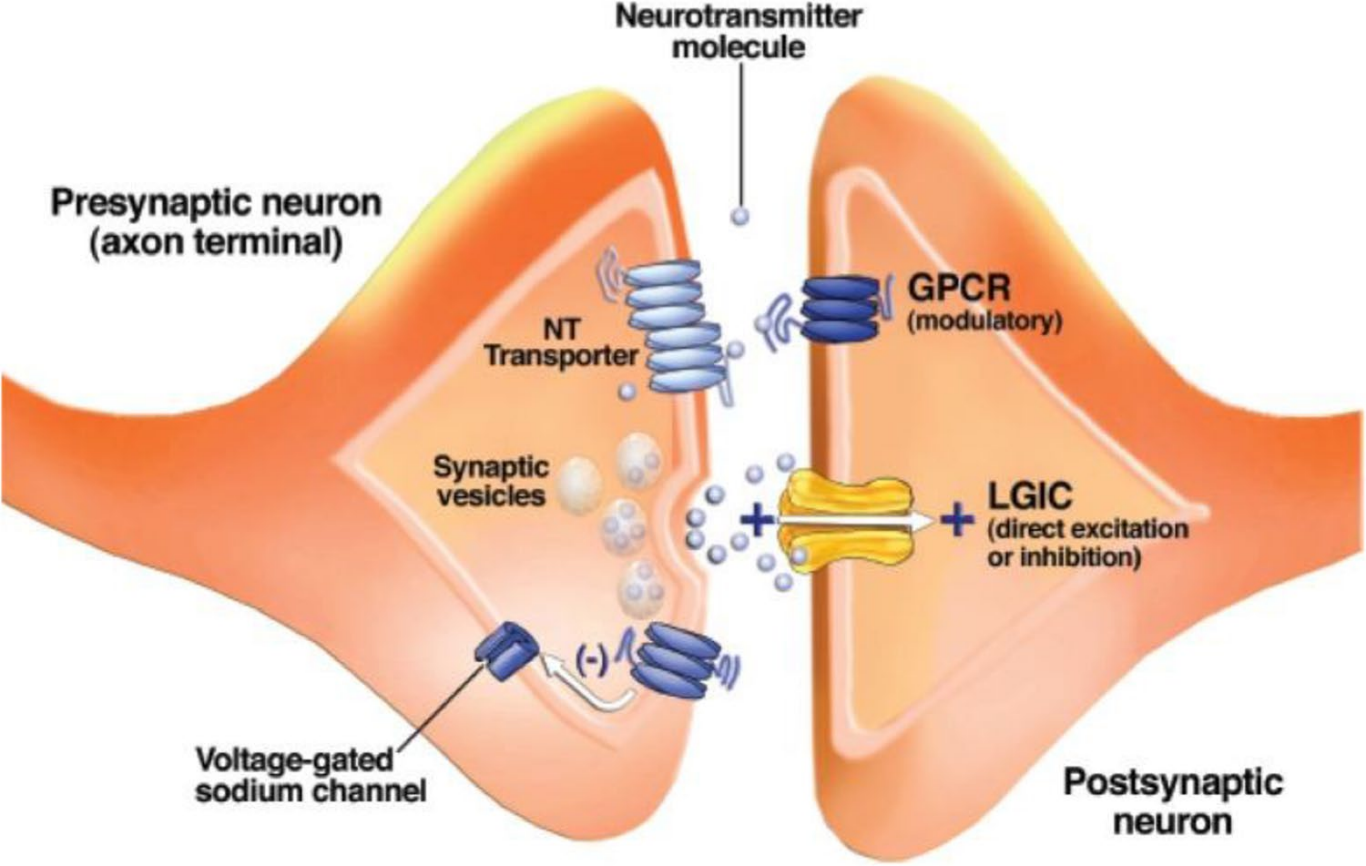
Schematic drawing of a synapse between two neurons. Synaptic vesicles contain a neurotransmitter (NT) and release it when their membranes fuse with the outer cell membrane. Neurotransmitter molecules cross the synaptic cleft and bind to receptors known as ligand-gated ion channels (LGICs) and G-protein–coupled receptors (GPCRs) on the postsynaptic neuron. GPCRs on the presynaptic neuron’s axon terminal alter the function of voltage-gated ion channels and modulate neurotransmitter release. Neurotransmitter transporters remove neurotransmitter molecules from the synaptic cleft so that they can be repackaged into vesicles. Reproduced with permission from [177]. 2008, The National Institute on Alcohol Abuse and Alcoholism

The communication network within the brain is a complex environment that involves electrical and chemical signals [176]. More specifically, represented in Fig. 6, in chemical signalling, a molecular signal to transmit, a receptor for transduction, and a target molecule for reception play specific roles [177].

Exploiting the application of stromal cell-derived factor-1α/C-X-C chemokine receptor 4 (SDF-1α/CXCR4) showed promising results [108, 152] since this chemokine and its CXCR4 and CXCR7 receptors are expressed by microglia, astrocytes, and vascular ECs in the CNS [178]. Its potential to drive endogenous neural progenitors cells has been studied in laminin (Lm)-based ECM [179]. Results proved the potential of SDF-1α to improve cellular migration and differentiation within the substrate. However, they found that proliferation was only dependent on SDF-1α independently to the substrate. Given that SDF-1α is upregulated following stroke [180], the same team developed a HA and Lm hydrogel to promote exogenous neural progenitor cells [181], with HA used to modulate CRCX4 [51], and Lm used to improve adhesion and cellular migration. The increase of CRCX4 in response to SDF-1α upregulation improved chemotactic signals for exogenous cell migration.

Caveolin-1 is also involved in signalling pathways and Gao et al. [182] demonstrated that when associated with VEGF, it promotes angiogenesis in a rat model through treadmill exercise. It should be noted that exercise after TBI has been shown to improve neuroplasticity, as evidenced by in vivo experiments in animals [115] and humans [183]. It was shown that caveolin-1 is induced by exercise, it regulates VEGF, and an increase in blood vessels density and a reduction in cavity volume were noticed [182], while Zhao et al. [184] have further demonstrated the role of caveolin-1 in neurogenesis suggesting that the signalling pathway induced by exercise is worth further investigation.

## Neuroimaging

Neuroimaging is a non-invasive methodology to target injected hydrogels, biomolecules, and cells in order to monitor the remodelling of tissue [172]. Several in vitro and in vivo imaging techniques are suitable for biomaterials imaging including MRI, positron emission tomography (PET), single photon emission computed tomography (SPECT), and others [185, 186]. Each imaging modality has strengths and limitations over the other, where MRI offers better spatial resolution of the scaffold, while PET-CT offers higher sensitivity for metabolic and functional activity within the scaffold [187]. Hence, the combination of multiple imaging modalities is sought to provide an accurate anatomical and highly sensitive synergistic quantitative data of the neural scaffolding tissue.

### Magnetic resonance imaging

MRI provides information about anatomical features of opaque tissues and has been extensively used for clinical diagnosis due to its non-invasive and non-radiative properties [188]. Moreover, it can be also employed for scaffold visualisation after its transplantation into the brain [185] for monitoring of the implanted biomaterials. Due to the high-water content of hydrogels, it is often hard to distinguish it from the adjacent brain tissue. To avoid this scenario, certain contrasting agents are employed [189]. For example, superparamagnetic iron oxide nanoparticles (SPIONs) have been reported to label induced-pluripotent stem (IPS) cells for tracking and visualisation after transplantation in an in vivo TBI rat model [190]. However, SPION labelling of stem cells also presents limitations. MRI signal rapidly decreases over time because of the rate of stem cell proliferation [191]. Moreover, MRI signal also declines due to the clearance of dead transplanted cell by phagocytic cells and exocytosis of SPION by surviving transplanted cells [192].

**Fig. 7:**
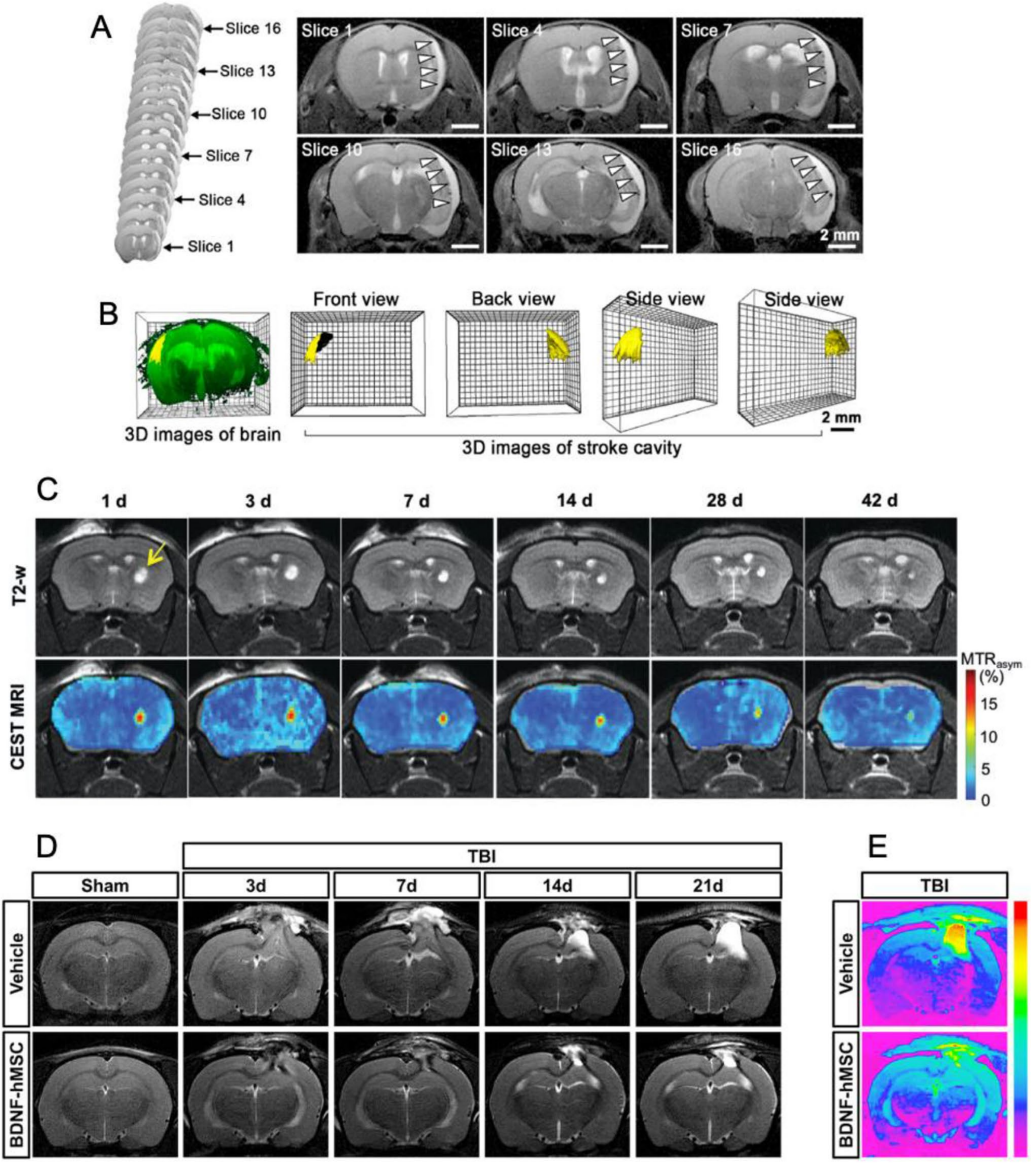
**A** The continuous MRI image series (left) covering the MCAO mouse’s brain for the in vivo application of a shape-customised CNTs-SS for filling a random stroke cavity. Six representative slices from these MRI images showed the site and shape of the stroke cavity (white arrowheads). **B** The 3D reconstruction (left) of MRI images of the MCAO mouse’s full brain (green) with the volumetrically rendered stroke cavity (yellow). **C** In vivo CEST MRI imaging of gelatin containing-HA hydrogel degradation. Time course of T2‐weighted and CEST MRI (at 3.6 ppm) from day 1 to day 42. Arrow indicates the region of hydrogel injection in the striatum. **D** Representative T2-weighted images from vehicle- or BDNF-hMSCs-treated rats 3, 7, 14, and 21 days after TBI. **E** Representative T2 maps of vehicle-treated or BDNF-hMSCs-treated rats 21 days after TBI. Brain oedema is represented by higher T2 value (in red). Reproduced with permission from **A** and **B** [194] 2021, Elsevier B.V, **C** [197], 2019, John Wiley & Sons, Inc, and **D** [208] 2020, Elsevier Ltd

3D structures of the brain can be obtained acquiring several MRI images of brain slices. This allows to develop a personalised treatment for the injured brain cavity, leading to a higher chance of regeneration success [187]. Fu et al. [193] produced personalised scaffolds which were cavity-specific. They produced a TBI model in adult male Sprague–Dawley rats by an electrically controlled cortical impactor. With a 3.0 T MRI scanner, in vivo images of rat brain were obtained to modulate the cavity produced after stroke simulation. This process allowed them to print, using a 3D Bioplotter, a collagen-chitosan scaffold that can be adapted to different brain defects [193]. Also to produce personalised scaffolds, Wang et al. [194] reported a work on carbon-nanotubes-doped sericin scaffold (CNTs-SS) which can be injected and has photoluminescent and programmable shape-memory properties. Figure 7A shows 6 slices of MRI of an MCAO mouse showing the stroke cavity and Fig. 7B the 3D reconstruction of the volumetrically rendered cavity, shown in yellow. The reconstruction of these images leads to a personalised fabrication of a shape-customised scaffold, after preparation of 3D-printed moulds for casting of CNTs-SS [194].

With the aim to study the degradation of a scaffold after a brain injury, Liang et al. [195] worked on the imaging of a label-free gelatin-containing HA hydrogel through chemical exchange saturation transfer (CEST) MRI [196] to study the changes of the hydrogel in vivo. The hydrogel was injected into the striatum of *rag2*^*−/−*^ mice model and the contrast properties of the hydrogel were evaluated. A strong CEST signal was observed due to the presence of gelation and an overall decrease in the signal after 7 days of implantation, meaning the hydrogel decomposed gradually [195]. Following this work, Zho et al. [197] monitored the biodegradation of the scaffold through CEST MRI in vivo, shown in Fig. 7C. As the hydrogel has a high-water content, the T2-weighted signal of the hydrogel was easily identified (Fig. 7C, top). CEST MRI signal is specific for the exchangeable protons within the hydrogel, showing a continuous decrease over 42 days (Fig. 7C, bottom) [197].

Often ‘naked’ hydrogel visualisation using MRI is not possible and labelling with MRI contrast agents is required [198]. Iron oxide [199], gadolinium (Gd) [200], fluorine (F) [201], and manganese (Mn) [202] are some of the most frequently used contrast agents. Gd is potentially toxic, this is why research is more focused on the use of iron oxide or Mn-based nanoparticles and materials for scaffold visualisation [203–205]. Vieira et al. [206] worked on a hydrogel based on methacrylated gellan gum and HA, and produced blends of hydrogels with paramagnetic Mn^2+^. These blends have allowed the real-time monitorisation of hydrogel deposition via T1-weighted MRI. Hydrogel degradation was also followed after in vitro and in vivo experimental approaches [206].

Not only is MRI useful for hydrogel visualisation but also allows for changes in the brain structure to be monitored. Diffusion tensor orientation is highly sensitive to microstructural brain alterations and it is potentially useful as a microstructural MRI biomarker for underlying neuropathologic changes after experimental and human TBI analysis [207]. Sultan et al. [208] have reported a hydrogel which reduces the oedema in brain after a TBI and used MRI imaging to demonstrate the reduced structural damage when this hydrogel is applied. A silk fibroin (SG) hydrogel with encapsulated human MSCs (hMSCs) was created to produce BDNF. A controlled cortical impact (CCI) model of experimental TBI [209] was performed on Sprague–Dawley male rats and the hydrogel was transplanted into the pocket created between the nasal septum and the mucosa. As a control, hydrogel without BDNF-hMSCs (vehicle) was used. Figure 7D shows T2-weighted images which demonstrate the reduced structural damage in rats transplanted with BDNF-hMSCs after 3, 7, 14, and 21 days post-TBI, compared with rats transplanted with the vehicle. The T2 map shown on Fig. 7E represents the oedema, in red [208].

### Computed tomography (CT)

In the evaluation of brain-associated injuries and abnormalities, CT imaging is used as the first line of investigation due to its availability and rapid image acquisition, in contrast to MRI. Owing to the clinical relevance of CT imaging, it is also a technique utilised to analyse tissue engineering constructs for CNS repair. For example, the visualisation of radiopaque hydrogels can be acquired through CT scans due to the variable absorption of X-rays (ionising radiation) by different tissues in the body [210]. CT imaging can demonstrate the microstructure and degradation profile of implanted biodegradable scaffolds implanted in the brain and spinal cord of mice [211, 212].

### Nuclear medicine imaging

PET-CT and SPECT are nuclear imaging techniques that combine the use of ionising radiation and radiotracers to label numerous cell types (including neural and stem cells) or biomaterials to obtain functional tissue information [213]. For example, biomaterials such as polytopic alginate can be cross-linked with several radiotracers for the in vivo visualisation of the hydrogel through SPECT or PET imaging [214]. More recently, genetically labelled cells using a reporter gene have been shown to withstand repetitive imaging. The genetically labelled cells pass on the reporting genes to their progeny which allows their observation through long periods of time [215]. For example, a PET reporter gene system using the pyruvate kinase (PKM2) gene with its associated radiotracer [^18^F]DASA-23 was able to be delivered by an adeno-associated virus to all areas of the CNS without breaking the blood–brain barrier for the monitoring of neural cell therapy [216]. It is expected that nuclear medicine imaging together with advances in reporter gene labelling will improve the functional and metabolic evaluation of tissue-engineered constructs for brain injury repair.

## Conclusion

The time of treatment for TBI patients is quintessential for their recovery. Recent studies have shown that interventional neuroradiology approaches within the first 60 min of symptom onset produce excellent outcomes in patients. This remarkable 1-h window from the onset of symptoms is known as The Golden Hour, where treatment within this timeframe will significantly lower rates of morbidity and mortality in patients. However, delayed diagnosis and treatment dramatically decrease patient recovery as tissue injury is irreversibly compromised. Thus, for these patients, the promotion of brain tissue healing through tissue engineering strategies is sought to improve neurogenesis at the site of injury and consequently leads to better motor function recovery outcomes. In vivo scaffold degradation and tissue repair and regeneration can be monitored through MRI. In this landscape, neuronal migration, angiogenesis, glial scar modulation, and signalling promoters are all important aspects to be consider in brain tissue healing for the development of a functional platform for brain tissue regeneration.

Tissue engineering strategies that combine the use of biomaterials and drug therapies are able to reduce inflammation, protect exogenous cells, and promote their differentiation in situ. However, scaffolding parameters such as biodegradability, swelling, porosity, pore size, topography, pore alignment, and modulus are also important to consider in order to develop a functional engineered brain tissue. The structural-function relationships between all these parameters will greatly impact cell function and dictate the final biological performance of the graft to mimic specific regions of the brain. Moreover, the scaffold- and cellular-based strategies highlighted in this review are sought to be applied in the development of reliable in vitro models of the human brain. The development of functional brain organoids is highly anticipated to improve in vitro studies and decrease the need of animal models in research for a better display of the connectivity and plasticity of the human brain.

## Data Availability

Data sharing not applicable to this article as no datasets were generated or analysed during the current review article.
